# Supplementary material for: Comparison of adult versus elderly patients with abdominal trauma: A retrospective database analysis
Source: PLoS One. 2024 Aug 19;19(8):e0309174. doi: 10.1371/journal.pone.0309174 (PMC11332922; doi:10.1371/journal.pone.0309174)
Supplement: Attachment — Submitted filename: Response to Reviewers.docx [file pone.0309174.s001.docx]

Jeremy Dash

Geneva University Hospitals

Rue Gabrielle-Perret-Gentil 4

1205 Geneva

Switzerland

Yusuke Tsutsumi

Academic Editor

PLOS ONE

The 24th of June 2024

Dear Yusuke Tsutsumi, dear Reviewers,

Thank you for the opportunity to revise and resubmit our manuscript, titled « Comparison of adult versus elderly patients with abdominal trauma: a retrospective database analysis ». We appreciate the reviewers’ valuable feedback and have addressed each of their comments in detail below.

**Journal Requirements:**

1*. Please ensure that your manuscript meets PLOS ONE's style requirements, including those for file naming. The PLOS ONE style templates can be found at https://journals.plos.org/plosone/s/file?id=wjVg/PLOSOne_formatting_sample_main_body.pdf and https://journals.plos.org/plosone/s/file?id=ba62/PLOSOne_formatting_sample_title_authors_affiliations.pdf*

Response :

- Continuous line numbers have been added.
- Page numbers appears now on the lower left hand corner.
- Figure citations have been corrected.
- Other manuscript body formatting guidelines have been double checked according to the modified April 2017 *Manuscript body formatting guidelines* document.
- Affiliation footnotes on title page has been modified according to the modified January 2017 *Title,author, affiliations formatting guidelines* document.

2. *We note that you have indicated that there are restrictions to data sharing for this study. For studies involving human research participant data or other sensitive data, we encourage authors to share de-identified or anonymized data. However, when data cannot be publicly shared for ethical reasons, we allow authors to make their data sets available upon request. For information on unacceptable data access restrictions, please see http://journals.plos.org/plosone/s/data-availability#loc-unacceptable-data-access-restrictions.*

Response :

We fully support the principles of transparency and data sharing in scientific research. However, the data used in our study includes sensitive information such as patient age, mechanism of trauma, year of the trauma, injuries and the length of stay in two recognizable hospitals. Due to the nature of these data points, there is a risk that individuals could be identifiable, thereby compromising patient confidentiality and privacy.

Our research adheres to stringent ethical guidelines and complies with the privacy regulations set forth by our institutions and the New Federal Act on Data Protection in Switzerland and the General Data Protection Regulation in France. These regulations impose strict limitations on the sharing of personal health information to protect patient privacy.

While we are unable to share the raw data publicly, we are committed to ensuring the transparency and reproducibility of our research. To this end, we can provide the contact information to which data requests may be sent :

**For the Geneva University Hospital registry :**

Dr Axel Gamulin

Hôpitaux Universitaires de Genève

Rue Gabrielle-Perret-Gentil 4

1211 Genève

axel.gamulin@hcuge.ch

0041223723311

Commission cantonale d'éthique de la recherche CCER

Rue Adrien-Lachenal 8

1207 Genève

0041 22 546 51 01

ccer@etat.ge.ch

www.ge.ch/lc/ccer

**For the Nice registry (REGISTRY CIL n°272) :**

Dr Damien MASSALOU

Centre Hospitalier Universitaire de Nice

Voie Romaine 30

CS 51069 - 06001 Nice Cedex 1

massalou.d@chu-nice.fr

0033663269264 / 0033492038614

Non-author point of contact :

Commission nationale de l'informatique et des libertés

3 Place de Fontenoy

TSA 80715

75334 PARIS CEDEX 07

0033153732222

**Review Comments to the Author**

Reviewer #1 :

*1. Why is there no data available from 2019 till present date keeping in mind that the number of patients in the elderly group is quite low and there are only short-term outcome paramters analyzed?*

Response :

We acknowledge the concern regarding the data timeframe and the size of the elderly patient group. The data presented in our study were collected in 2020. At that time, we had access to patient records up until the end of 2019. Unfortunately, due to institutional and administrative constraints, we did not have access to more recent data beyond 2019 during our study period. We believe the revisions and responses provided have significantly improved the manuscript. We hope the reviewers find our responses satisfactory and that the manuscript is now suitable for publication.

We appreciate your interest in longer-term outcome parameters. The scope of our study was limited by the information available in our registries, which did not contain long-term follow-up data. Our registries are primarily designed for short-term outcome tracking, which includes immediate and early postoperative results. Unfortunately, they do not extend to longer-term outcomes.

We understand the importance of long-term data in providing a more comprehensive view of patient outcomes. As part of our future research initiatives, we plan to enhance our data collection methods to include longer-term follow-up information, provided we can secure the necessary resources and ethical approvals.

*2. Data on the pattern of abdominal trauma is missing (e. g. how many splenic, hepatic or renal injuries appeared in the corresponding groups)*

Response :

We have added the relevant data in the results section of the manuscript. The data regarding the number of patients suffering from splenic, hepatic and renal injuries and the occurrence of splenectomy and embolisation in both the elderly and younger groups, and the corresponding statistical analysis have been detailed.

*3. -If data from certain parameters like vital signs are missing and patients were included in the study anyways, a number of patients analyzed for each group and paramter should be given (e. g. how many patients were analyzed regarding systolic blood pressure in the YG an EG).*

We added in the method paragraph the number of patients with missing values.

Reviewer #2 :

*1. Thank you for giving me the opportunity to review this interesting manuscript. This theme is very important. Your manuscript is well written. So I have only one comment.

Methods:
Not multivariate but multivariable analyses. It is the same for the after section.
“The multivariate analyses was adjusted to the following factors (age, penetrating injuries, shock, GCS, SBP, DBP, hb, arterial lactates, abdominal and thoracic AIS and ISS). We used the R statistical software to perform statistical analysis.”
Hidalgo B, Goodman M. Multivariate or Multivariable Regression? Am J Public Health. 2013;103(1):39-40. doi:10.2105/AJPH.2012.300897*

Response :

We have corrected " -variate " to " -variable ". Thank you for your comment and for recommending the interesting article that explains the difference and highlights the confusion between the two terms.

Thank you for your consideration.

Sincerely,

Jeremy Dash
