# Supplementary material for: Comparison of adult versus elderly patients with abdominal trauma: A retrospective database analysis
Source: PLoS One. 2024 Aug 19;19(8):e0309174. doi: 10.1371/journal.pone.0309174 (PMC11332922; doi:10.1371/journal.pone.0309174)
Supplement: Attachment — Submitted filename: Response to Reviewers.docx [file pone.0309174.s002.docx]

Jeremy Dash

Geneva University Hospitals

Rue Gabrielle-Perret-Gentil 4

1205 Geneva

Switzerland

Yusuke Tsutsumi

Academic Editor

PLOS ONE

The 5th of August 2024

Dear Yusuke Tsutsumi, dear Reviewers,

Thank you for the opportunity to revise and resubmit our manuscript, titled « Comparison of adult versus elderly patients with abdominal trauma: a retrospective database analysis ». We appreciate the reviewers’ valuable feedback and have addressed each of their comments in detail below.

**Journal Requirements:**

1*. Please review your reference list to ensure that it is complete and correct. If you have cited papers that have been retracted, please include the rationale for doing so in the manuscript text, or remove these references and replace them with relevant current references. Any changes to the reference list should be mentioned in the rebuttal letter that accompanies your revised manuscript. If you need to cite a retracted article, indicate the article’s retracted status in the References list and also include a citation and full reference for the retraction notice.*

Response :

All references have been reviewed.

Reference number 8 *(Sasser SM, Hunt RC, Faul M, Sugerman D, Pearson WS, Dulski T, Galli RL. Guidelines for field triage of injured patients: recommendations of the National Expert Panel on Field Triage, 2011. Morbidity and Mortality Weekly Report: Recommendations and Reports. 2012 Jan 13;61(1):1-20)* : we noted that there is a more recent version reported by The American College of Surgeons. However there is no modification regarding the recommandation that patients over 65 with systolic blood pressure under 110 mmHg should be considered at high risk for serious injury and transported to the highest-level trauma centre.

The updated reference is : *Newgard CD, Fischer PE, Gestring M, Michaels HN, Jurkovich GJ, Lerner EB, Fallat ME, Delbridge TR, Brown JB, Bulger EM. National guideline for the field triage of injured patients: recommendations of the National Expert Panel on Field Triage, 2021. Journal of Trauma and Acute Care Surgery. 2022 Aug 1;93(2):e49-60.*

All other references have been checked and none has been retracted.

**Additional Editor Comments:**

*1. I found the lack of recent data was important problem, while I understand the restricted access to the data. Therefore, I strongly recommend to describe how this lack of up-to-date data affects the results in the Limitation section.*

Response :

We acknowledge that the restricted access to more current data is an important issue, and we have discussed how this limitation may affect the results and their applicability to current clinical practices in the Limitations section.

*2.I think the manuscript have been much improved by including details of abdominal trauma in Table 3. However, the contents of Table 3 are inconsistent. For spleen injury, there is the information about the treatment (splenectomy, embolization). However, for the other two injuries, there is no information about treatments. For kidney injury, there is no information of AAST score. Please revise the contents.*

Response :

The content has been revised and we added the missing values and discussed the results.

*3. Additionally, the layout of Table 3 is confusing, in which, injury site, AAST score and treatments are all arranged as same level of rows. Please revise the design of Table 3 using sub-headings.*

Response :

The design of Table 3 has been changed using sub-headings. Missing value have been added.

*4. As just a minor point, I think the last table is Table 5 not Table 4 (there are two Table 4s). Please revise.*

Response :

Correction has been made.

Thank you for your consideration and for your valuable reviewing.

Sincerely,

Jeremy Dash
